# Surface Immune Checkpoints as Potential Biomarkers in Physiological Pregnancy and Recurrent Pregnancy Loss

**DOI:** 10.3390/ijms25179378

**Published:** 2024-08-29

**Authors:** Michał Zych, Monika Kniotek, Aleksander Roszczyk, Filip Dąbrowski, Robert Jędra, Radosław Zagożdżon

**Affiliations:** 1 Department of Clinical Immunology, Medical University of Warsaw, Nowogrodzka 59, 02-006 Warsaw, Poland; michal.zych27@gmail.com (M.Z.); aleksander.roszczyk@gmail.com (A.R.); 2 Department of Gynecology and Gynecological Oncology, Medical Centre of Postgraduate Medical Education, CMKP, Marymoncka 99/103, 01-813 Warsaw, Poland; fil.dabrowski@gmail.com (F.D.); robert.jedra@gmail.com (R.J.); 3 Club35, Polish Society of Obstetricians and Gynecologists PTGiP, Cybernetyki7F/87, 02-677 Warsaw, Poland; 4 Laboratory of Cellular and Genetic Therapies, Medical University of Warsaw, Banacha 1B, 02-097 Warsaw, Poland; radoslaw.zagozdzon@wum.edu.pl

**Keywords:** immune checkpoints, miscarriage, pregnancy loss, PD-1, RSA, recurrent spontaneous abortion, TIM-3, TIGIT, VISTA

## Abstract

Due to the genetic diversity between the mother and the fetus, heightened control over the immune system during pregnancy is crucial. Immunological parameters determined by clinicians in women with idiopathic recurrent spontaneous abortion (RSA) include the quantity and activity of Natural Killer (NK) and Natural Killer T (NKT) cells, the quantity of regulatory T lymphocytes, and the ratio of pro-inflammatory cytokines, which indicate imbalances in Th1 and Th2 cell response. The processes are controlled by immune checkpoint proteins (ICPs) expressed on the surface of immune cells. We aim to investigate differences in the expression of ICPs on T cells, T regulatory lymphocytes, NK cells, and NKT cells in peripheral blood samples collected from RSA women, pregnant women, and healthy multiparous women. We aim to discover new insights into the role of ICPs involved in recurrent pregnancy loss. Peripheral blood mononuclear cells (PBMCs) were isolated by gradient centrifugation from blood samples obtained from 10 multiparous women, 20 pregnant women (11–14th week of pregnancy), and 20 RSA women, at maximum of 72 h after miscarriage. The PBMCs were stained for flow cytometry analysis. Standard flow cytometry immunophenotyping of PBMCs was performed using antibodies against classical lymphocyte markers, including CD3, CD4, CD8, CD56, CD25, and CD127. Additionally, ICPs were investigated using antibodies against Programmed Death Protein-1 (PD-1, CD279), T cell immunoglobulin and mucin domain-containing protein 3 (TIM-3, CD366), V-domain Ig suppressor of T cell activation (VISTA), T cell immunoglobulin and ITIM domain (TIGIT), and Lymphocyte activation gene 3 (LAG-3). We observed differences in the surface expression of ICPs in the analyzed subpopulations of lymphocytes between early pregnancy and RSA, after miscarriage, and in women. We noted diminished expression of PD-1 on T lymphocytes (*p* = 0.0046), T helper cells (CD3CD4 positive cells, *p* = 0.0165), T cytotoxic cells (CD3CD8 positive cells, *p* = 0.0046), T regulatory lymphocytes (CD3CD4CD25CD127 low positive cells, *p* = 0.0106), and NKT cells (CD3CD56/CD16 positive cells, *p* = 0.0438), as well as LAG-3 on lymphocytes T (*p* = 0.0225) T helper, *p* = 0.0426), T cytotoxic cells (*p* = 0.0458) and Treg (*p* = 0.0293), and cells from RSA women. Impaired expression of TIM-3 (*p* = 0.0226) and VISTA (*p* = 0.0039) on CD8 cytotoxic T and NK (TIM3 *p* = 0.0482; VISTA *p* = 0.0118) cells was shown, with an accompanying increased expression of TIGIT (*p* = 0.0211) on NKT cells. The changes in the expression of surface immune checkpoints indicate their involvement in the regulation of pregnancy. The data might be utilized to develop specific therapies for RSA women based on the modulation of ICP expression.

## 1. Introduction

The establishment of immunological tolerance within the maternal immune system towards fetal cells is a crucial component of successful pregnancy. A physiologically operational maternal immune system acknowledges the presence of paternal antigens presented by the fetus but refrains from initiating an immune response. This finely tuned operation necessitates stringent control through various regulatory mechanisms on multiple levels. Any disruptions in this delicate balance can result in self-tissue damage, leading to complications in pregnancy, including recurrent spontaneous abortions (RSAs). Despite its significance, the correlation between the maternal immune system and pregnancy development remains an area with limited exploration. In this work, we have primarily focused on co-inhibitory receptors that play pivotal roles in controlling effector immune cells and in shielding the organism against a detrimental immune response that targets self-antigens. 

### 1.1. Programmed Cell Death Protein 1 (PD-1) 

Programmed cell death protein 1 (PD-1) belongs to the B7-CD28 family of molecules. The transmembrane protein is expressed on the surface of a diverse range of activated immune cells, such as B cells, T helper cells, T cytotoxic lymphocytes, regulatory T cells, Natural Killer T cells, monocytes, and dendritic cells [1,2,3]. PD-1 blocks functional T cells in the late immune response [1]. 

There are two known ligands for PD-1, Programmed Cell Death Ligand 1 (PD-L1) and Programmed Cell Death Ligand 2 (PD-L2). Both ligands are members of the B7 family [2]. PD-L1 is expressed on antigen-presenting cells (APCs) and various cell types including cardiomyocytes, vascular endothelium cells, hepatocytes, keratinocytes, and placental syncytiotrophoblasts [3]. PD-L1’s presence in the periphery protects tissues from autoimmune damage [3,4]. PD-L2 expression is limited to macrophages and dendritic cells [2,3]. The dissemination of PD-1 ligands plays a role in establishing tolerance in peripheral tissues [3,5].

Induction of tolerance and regulation of local inflammation is achieved by engaging PD-1 with its ligands [6]. PD-1 ligation inhibits PI3K activity, which results in the activation of Akt and the subsequent inhibition of the production of proinflammatory cytokines such as IL-2 or IFN-γ [1,7]. Originally, PD-1 was described as a receptor on exhausted cells, which cannot receive stimulatory signals [3,8]. Overexpression of PD-1 results in T cell dysfunction and exhaustion, accompanied by impaired IL-2 and IFN-γ secretion through inhibiting the expression of the transcription factors GATA-3, T bet, and Eomes [1,2,6,7]. CD8^+^ lymphocytes overexpressing PD-1 are not able to proliferate and produce cytokines [1]. PDL–PD-1 interactions arrest cells in the G_0_/G_1_ phase but do not increase cell death [9]. Interactions between PD-1 and PD-L2 on CD4^+^ T cells dramatically inhibit (TCR)-mediated proliferation and cytokine production. PD-1 is responsible for the differentiation of naïve T cells into Treg cells, thus limiting the response of effector cells [10]. The connection of PD-1 with ligands induces peripheral tolerance [11]. The PD-1/PD-L1 pathway plays an important role in the regulation of immune suppression via reduced T cell proliferation, cell anergy and exhaustion, diminished cytokine production, and increased Treg function [12]. Indeed, data showed PD-L1 expression on the outer side of the syncytiotrophoblast layer but not on the inner side, and it was absent on the cytotrophoblast [13]. Concerning the above results, we can assume that the PD1/PD-L1 pathway is triggered in the maternal–fetal interphase and regulates immune tolerance mechanisms toward the fetus [11]. 

### 1.2. T Cell Immunoglobulin and Mucin Domain-Containing Protein 3 (TIM-3)

T cell immunoglobulin and mucin domain-containing protein 3 (TIM-3) is affecting of immune response in autoimmunity and cancer. TIM-3 expression was reported on various immune cell types: helper T cells, cytotoxic T cells, Natural Killer cells, mast cells, regulatory T cells, APC cells, and myeloid cells [14]. The connection of TIM-3 with its ligands triggers the phosphorylation of Tyr256 and Tyr2263 by the tyrosine kinase ITK and the release of BAT-3 [14,15]. The relaxation allows TIM-3 to exert its inhibitory function [14]. Potentially, tyrosine kinase FYN may bind to the same sites as BAT3 and induce T cell anergy by activating phosphoprotein associated with glycosphingolipid-enriched microdomains 1 (PAG). The activation results in the recruitment of the tyrosine kinase CSK, which subsequently phosphorylates LCK on an inhibitory residue, leading to the suppression of T cell receptor (TCR) signaling [14,16]. 

The first known and most important ligand in fetal immune tolerance attributed to TIM-3 is Galectin-9. Gal-9 is a C-type lectin that is expressed or secreted by numerous hematopoietic cells and endometrial tissue. Gal-9 binds to the immunoglobulin V domain of TIM-3 and induces the release of BAT3 from the intracellular tail. This reaction prompts intracellular calcium influx, causing inactivation and T cell death [17]. Gal-9 serves as an endometrial marker for the mid- and late-secretory decidual phases. The expression of Gal-9 during the proliferative and early decidual phases is notably low [18,19]. dNKTIM-3^+^ cells exhibiting an immunosuppressive phenotype constitute the predominant subset of decidual immune cells [14,20]. Robust interaction between dNKTIM-3+ cells and Gal-9 has been identified as a prerequisite for the establishment of physiological pregnancy [21]. FOXP-3+TIM-3+ Treg cells demonstrate enhanced suppressive functionality attributed to their abundance in effector mechanisms such as IL-10, granzyme A, or perforin [20,22].

TIM-3 has been documented to exert a negative regulatory effect on immune responses during viral infections [21]. It plays a crucial role in preventing autoimmunity in organisms. Diminished surface expression of TIM-3 on T cells has been observed in patients with ulcerative colitis, multiple sclerosis, or rheumatoid arthritis [23,24,25,26,27].

### 1.3. Lymphocyte Activation Gene-3 (LAG-3) 

Lymphocyte activation gene-3 (LAG-3) is a type I transmembrane protein that shares structural similarities with CD4 [28]. Currently, LAG-3, alongside PD1, stands as a primary candidate for cancer therapy [28]. The structure of LAG-3 consists of four Ig-like domains: D1–D4. Within the LAG-3 cytoplasmic tail, three distinct regions are identified: the first harbors a serine phosphorylation site, the second contains a unique KIEELE motif, and the third comprises glutamic acid–proline (EP) repeats [29]. The exact signaling pathway for LAG-3 remains unclear. While the extracellular region of LAG-3 shares approximately 20% amino acid homology with CD4, it lacks a palmitoylation site found in CD4 and lacks cysteine residues necessary for association with lymphocyte-specific protein tyrosine kinase (Lck) [20,30,31,32,33]. LAG-3 expression on activated T cells is augmented by IL-2, IL-7, and IL-12 [34,35].

To date, ligands for LAG-3 have not been fully explored. Some studies show that Galectin-3 or liver sinusoidal endothelial cell lectin (LSECtin) may interact with LAG-3 [35,36]. Major histopathology complex II (MHC II) was proposed as a ligand for LAG-3. There was a theory that LAG-3 competes with CD4 for binding with MHC II, thereby causing lymphocyte inhibition. Further studies demonstrated that LAG-3 inhibits T cell activation through mechanisms distinct from the competitive inhibition of CD4 [37,38,39]. Recently, the MHCII transactivator (CIITA) has been identified as a critical regulator of the LAG-3 ligand. CIITA induces the expression of MHCII and MHCII accessory molecules, including CD74 and H2-DM. The MHCII accessory molecules are responsible for the formation and cell surface sorting of peptide–MHCII complexes (pMHCII), with a stable structural conformation in the conventional pathway of antigen presentation [40]. LAG-3 distinguishes the conformation of pMHCII and selectively binds to stable pMHCII. Accordingly, LAG-3 preferentially inhibits the activation of CD4^+^ T cells that recognize stable pMHCII [41]. It has also been demonstrated that LAG-3 does not compete with CD4 for pMHCII binding [28,38]. Wang et al. proposed fibrinogen-like protein 1 (FGL1), a member of the fibrinogen family, as a potential ligand for LAG-3 [41]. FGL1 is generally produced by hepatocytes in physiological conditions and by tumor cells [28,32]. 

LAG-3’s inhibitory function is correlated with its expression level on the surface of cells. It is expressed on T helper and T cytotoxic lymphocytes, NK cells, and CD4^+^ T regulatory lymphocytes [42]. Antigen stimulation leads to extensive expression of LAG-3. During chronic infection, LAG-3 is highly and sustainably expressed, and the state causes lymphocyte exhaustion [43,44]. LAG-3 promotes Treg cell-mediated suppression [35]. Blockade or elimination of LAG-3 in knockout mice promotes the development of Type 1 diabetes [37,45,46]. 

### 1.4. T Cell Immunoglobulin and ITIM Domain (TIGIT)

Firstly, this molecule was described by a bioinformatics algorithm as a new member of the CD28 family and was named VSIG9. Over time, it was renamed to VSTM3 and finally to TIGIT—T cell immunoglobulin and ITIM domain [32,47]. The receptor of the Ig superfamily is expressed in activated T cells, NK cells, memory T cells, and T regulatory cells [47,48,49,50].

Two ligands for TIGIT, CD155 (PVR) and CD112 (PVRL2, nectin-2), are expressed on APCs, T lymphocytes, and on non-hematopoietic cell types including tumor cells [32,49]. CD155 binds with higher affinity to TIGIT than CD112 [32]. TIGIT/CD226 and CD96 bind with similar affinity. CD226 associates with the integrin LFA-1 and delivers a positive signal, while TIGIT and CD96, after binding with CD155 and ITIM motifs in their cytoplasmic tails, deliver inhibitory signals. TIGIT not only competes with CD226 for ligands but in some cases, binds to CD226 and transmits a costimulatory signal [51]. TIGIT is structurally similar to the PVR-nectin family of proteins. This co-inhibitory molecule is composed of an extracellular IgV domain, a type 1 transmembrane region, a cytoplasmic tail containing ITIM, and an immunoglobulin tail tyrosine (ITT)-like motif [45,46,51]. Phosphorylation of a tyrosine residue in either the ITIM motif (Y231) or ITT-like motif (Y225) seems to be responsible for the inhibitory function of TIGIT [52,53]. When CD155 binds to TIGIT, it induces phosphorylation through the Fyn and Lck kinases and the recruitment of SHIP1 (SH2 domain-containing inositol-5-phosphatase (1)) through the cytosolic adaptor Grb2 (growth factor receptor-bound protein (2)). The recruitment of SHIP1 to the TIGIT tail inhibits signal transduction pathways involving PI3K (phosphoinositide 3-kinase) and MAPK kinases, resulting in the suppression of NK cell function [32,53,54]. Upon phosphorylation, the ITT-like motif of TIGIT binds β-arrestin 2 and recruits SHIP1 to limit NF-κB (nuclear factor-κB) signaling [29]. These pathways cause a reduction in NK cytotoxicity and cytokine secretion [52,53,54]. The activation of TIGIT in T cells has a significant impact on pathways regulating TCR components, including TCRα and CD3ε [44]. The activation of TIGIT is related to the induction of anti-apoptotic molecules such as Bcl-xL and the upregulation of receptors for IL-2, IL-7, and IL-15 as this action promotes T cell survival [55]. Engagement of CD155 on DCs with TIGIT induces the immunosuppressive function of DCs by triggering IL-10 production while decreasing IL-12 secretion and CD86 expression [56]. In turn, this indirectly inhibits T-cell function. Activation of TIGIT enhances Treg-mediated suppression through the secretion of IL-10 and Fibrinogen-Like 2 (FGL2), as well as the suppression of Th1/Th17 responses and the inhibition of Th1 activation [57].

### 1.5. V-Domain Ig-Containing Suppressor of T Cell Activation (VISTA) 

V-domain Ig-containing Suppressor of T cell Activation (VISTA), also known as PD-1H or B7-H5, is a type I transmembrane protein consisting of a single N-terminal immunoglobulin (Ig) V domain. VISTA was detected on the surface of macrophages, dendritic cells, circulating neutrophils, T helper cells (naïve and memory), Treg cells, T cytotoxic cells, and NK cells. VISTA is built up from approximately 279 amino acids. Its extracellular domains consist of 162 amino acids (aa), its transmembrane domain consists of 21 amino acids, and it has a 96 aa cytoplasmic tail [58,59]. The cytoplasmic domain is deprived of immunoreceptor tyrosine-based signaling motifs but possesses potential sites for protein C and casein kinase 2 phosphorylation sites [58,59,60]. Genetic analysis of VISTA showed similarities with PD-1, CD28, and CTLA-4, with the highest similarity to PD-1; however, analysis of the IgV domain of VISTA revealed the highest homology to PD-L1 [61]. Data on VISTA’s structure suggest that the molecule may act as both a ligand and a receptor [48]. 

## 2. Results

There were no differences between the age and BMI of the women enrolled in the study. The groups of women differed significantly in the number of miscarriages, term pregnancies, and duration of pregnancy due to specific selection into the groups, see Table 1.

**Figure 1:**
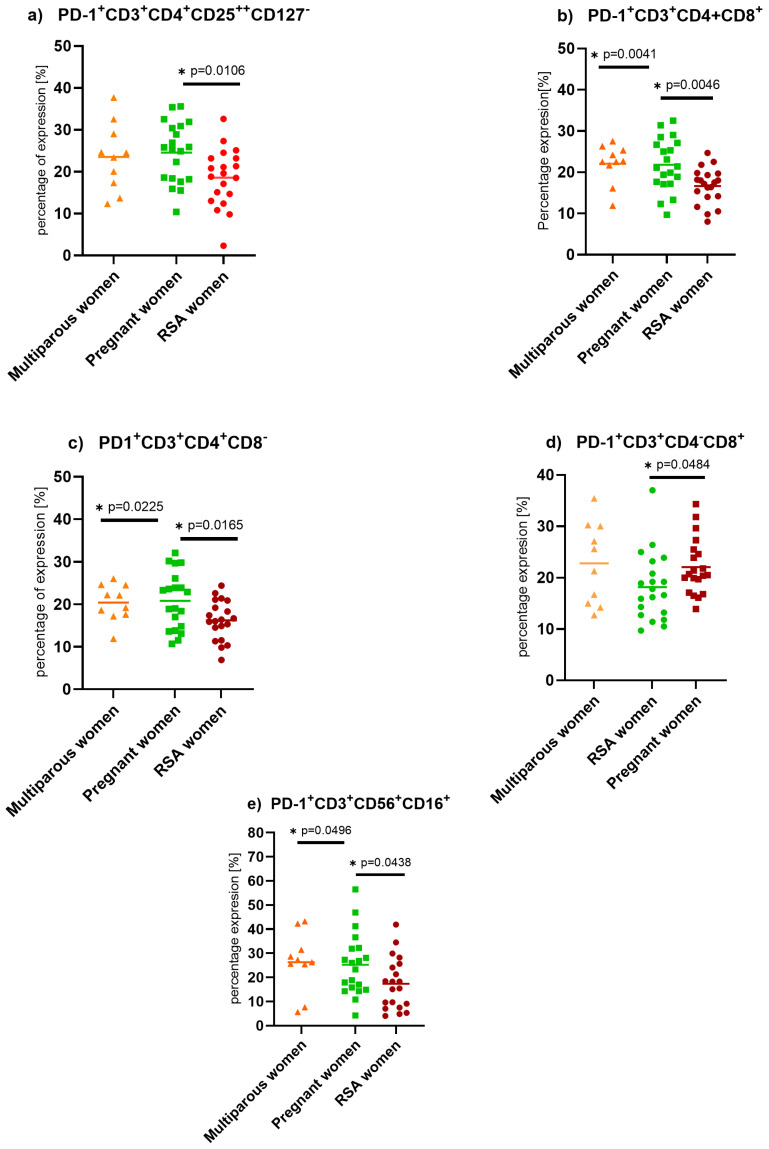
PD-1 expression on isolated PBMC lymphocytes in a control group of non-pregnant multiparous women (*n* = 10), pregnant women (*n* = 20), and RSA women (*n* = 20). Statistically significant differences between groups were considered when the “*p*” value was below 0.05 and are marked by a star. (**a**) PD-1 expression on Treg cells, (**b**) PD-1 expression on T lymphocytes, (**c**) PD-1 expression on T helper lymphocytes, (**d**) PD-1 expression on cytotoxic T lymphocytes, (**e**) PD-1 expression on NKT-like cells.

We found decreased expression of Programmed death protein–1 (PD-1) on helper (Wald_1_ = 5.733, *p* = 0.017, Exp(B) = 1.173), cytotoxic (Wald_1_ = 7.292, *p* = 0.007, Exp(B) = 1.158), regulatory T (Wald_1_ = 4.488, *p* = 0.034, Exp(B) = 0.726), and NKT cells (Wald_1_ = 4.007, *p* = 0.045, Exp(B) = 1.062) in RSA women compared to pregnant women, as well as impaired expression of PD-1 on NKT cells (Wald_1_ = 3.639, *p* = 0.056, Exp(B) = 1.070) when compared to non-pregnant women, Figure 1.

**Figure 2:**
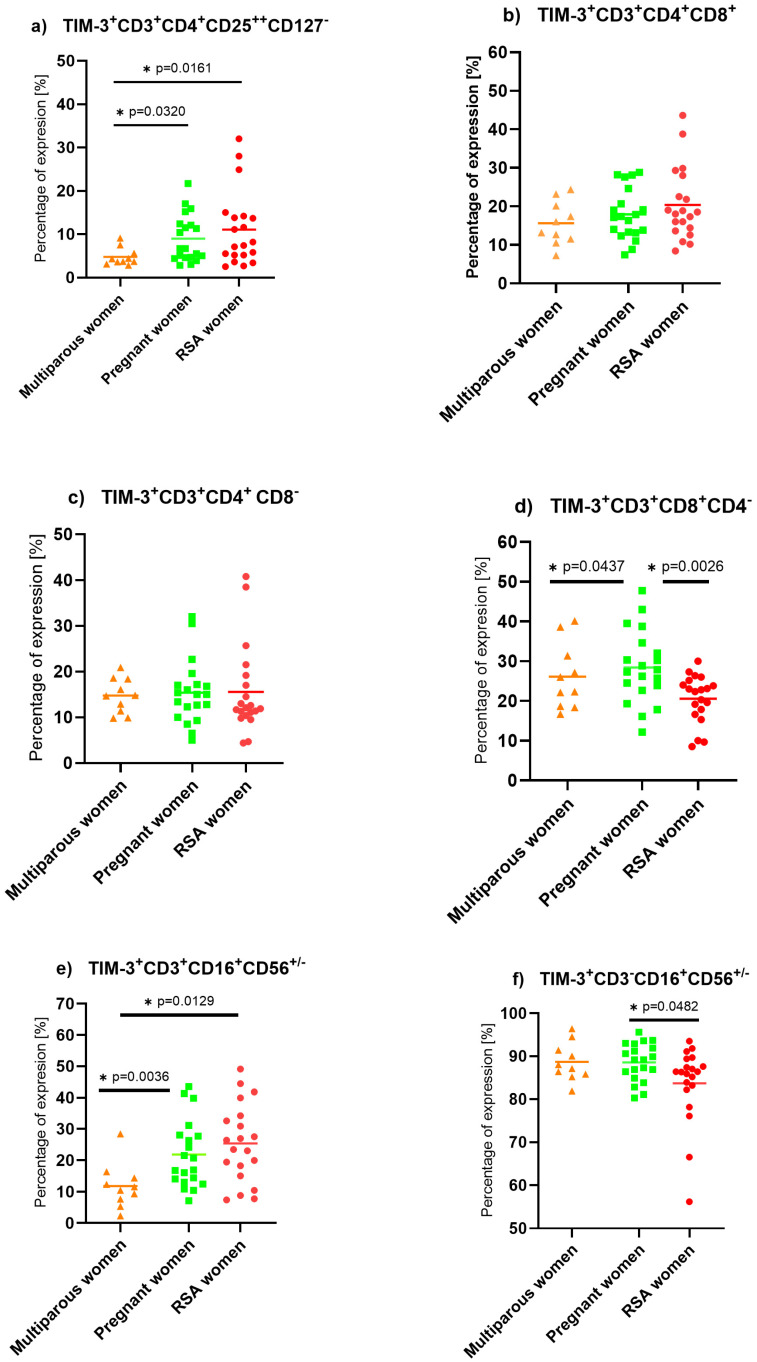
TIM-3 expression on isolated PBMC lymphocytes in a control group of non-pregnant multiparous women (*n* = 10), pregnant women (*n* = 20), and RSA women (*n* = 20). Statistically significant differences between groups were considered when the “*p*” value was below 0.05 and marked by a star. (**a**) TIM-3 expression on Treg cells, (**b**) TIM-3 expression on T lymphocytes, (**c**) TIM-3 expression on T helper lymphocytes, (**d**) TIM-3 expression on cytotoxic T lymphocytes, (**e**) TIM-3 expression on NKT-like cells, (**f**) TIM-3 expression on NK cells.

We observed decreased expression of TIM-3 on cytotoxic T lymphocytes (Wald_1_ = 7.292, *p* = 0.007, Exp(B)1.158) when comparing the lymphocytes of RSA to pregnant women. In contrast, the highest expression of TIM-3 on NKT (RSA vs. pregnant: Wald_1_ = 6.810, *p* = 0.009, Exp(B) = 0.847) and T reg (RSA vs. pregnant: Wald_1_ = 4.488, *p* = 0.034, Exp(B) = 0.726) cells was observed in the RSA group. The lowest occurrence of TIM- 3 was on multiparous NK lymphocytes, Figure 2.

The lowest expression of LAG-3 in the entire studied cell population was found among RSA women. Comparisons between non-pregnant and RSA women showed the following results:

(a) Treg cells: Wald1 = 10.178, *p* = 0.001, Exp(B) = 1.145

(b) NKT cells: Wald1 = 10.000, *p* = 0.002, Exp(B) = 1.108

(c) CD3 cells: Wald1 = 13.177, *p* < 0.001, Exp(B) = 1.185

(d)NK cells: Wald1 = 9.651, *p* = 0.002, Exp(B) = 1.143

**Figure 3:**
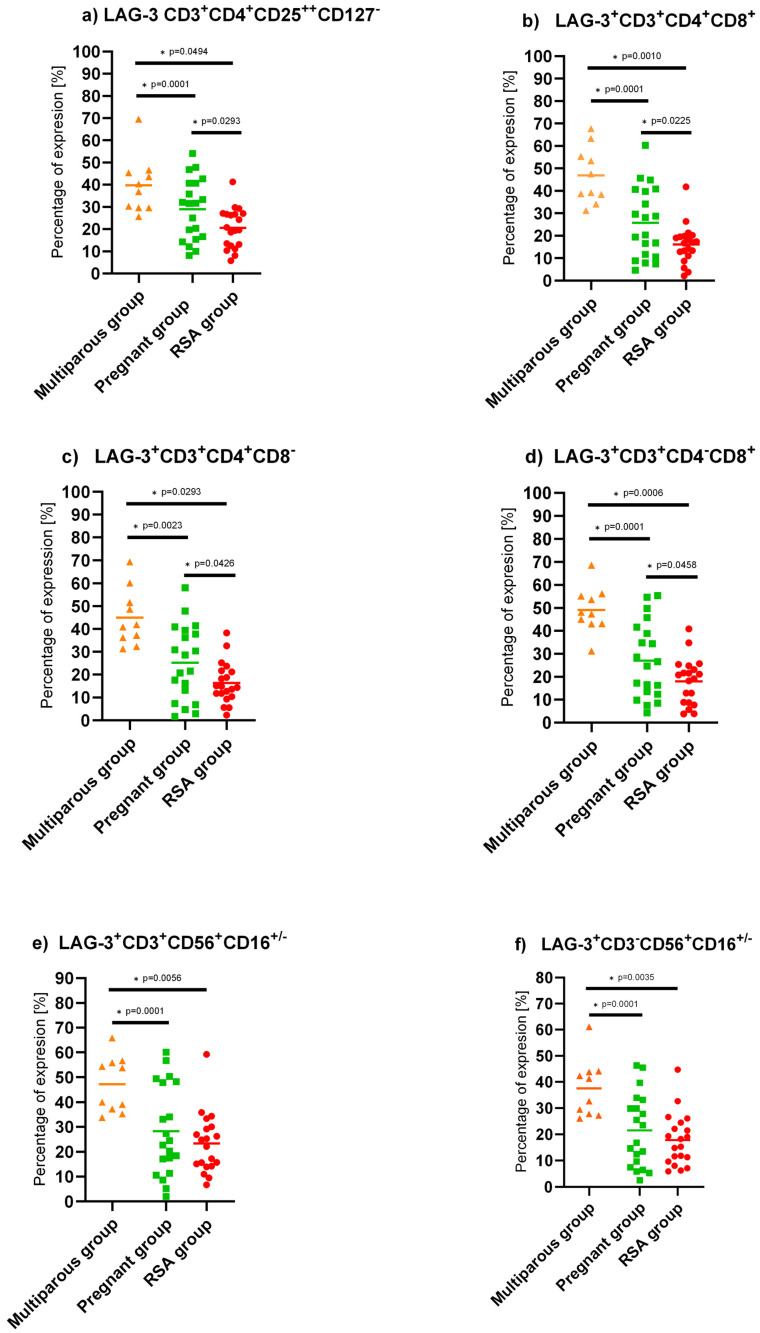
LAG-3 expression on isolated PBMC lymphocytes in a control group of non-pregnant multiparous women (*n* = 10), pregnant women (*n* = 20), and RSA women (*n* = 20). Statistically significant differences between groups were considered if “*p*” was below 0.05 and marked by a star. (**a**) LAG-3 expression on Treg cells, (**b**) LAG-3 expression on T lymphocytes, (**c**) LAG-3 expression on T helper lymphocytes, (**d**) LAG-3 expression on cytotoxic T lymphocytes, (**e**) LAG-3 expression on NKT-like cells, (**f**) LAG-3 expression on NK cells.

(e) CD4 cells: Wald1 = 11.981, *p* < 0.001, Exp(B) = 1.174

(f) CD8 cells: Wald1 = 12.072, *p* < 0.001, Exp(B) = 1.192. (Figure 3)

In the pregnant group, LAG-3 expression was decreased compared to the non-pregnant group in helper, cytotoxic, regulatory T lymphocytes, NK, and NKT cells. In the RSA group compared to the pregnant group, LAG-3 expression was significantly impaired in Treg cells (Wald1 = 4.654, *p* = 0.031, Exp(B) = 1.070) and CD3 cells (Wald1 = 4.654, *p* = 0.031, Exp(B) = 1.070), Figure 3.

**Figure 4:**
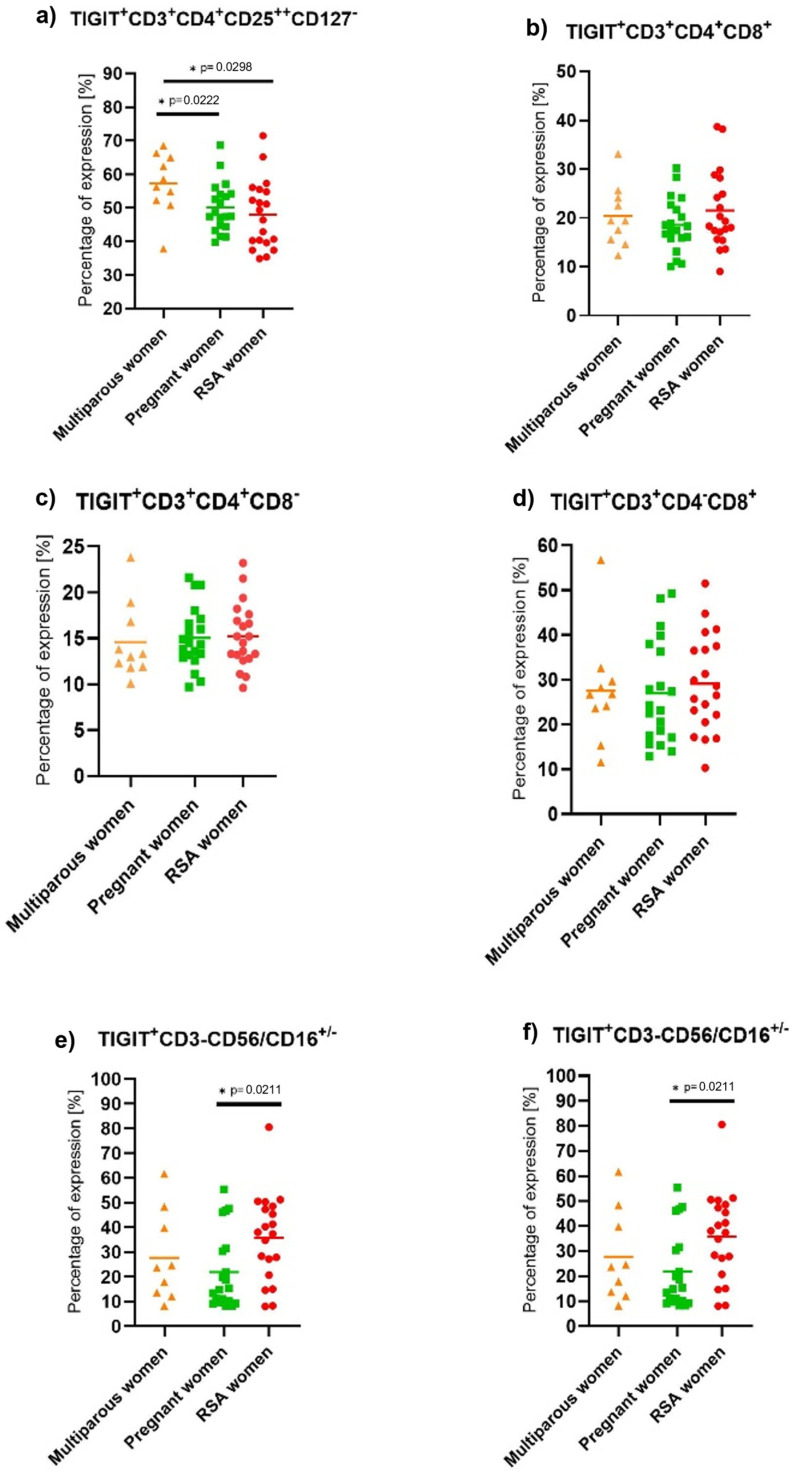
TIGIT expression on isolated PBMC lymphocytes in a control group of non-pregnant multiparous women (*n* = 10), pregnant women (*n* = 20), and RSA women (*n* = 20). Statistically significant differences between groups were considered if “*p*” was below 0.05 and marked by a star. (**a**) TIGIT expression on Treg cells, (**b**) TIGIT expression on T lymphocytes, (**c**) TIGIT expression on T helper lymphocytes, (**d**) TIM-3 expression on cytotoxic T lymphocytes, (**e**) TIGIT expression on NKT-like cells, (**f**) TIGIT expression on NK cells.

TIGIT expression was decreased in regulatory T lymphocyte subpopulation in both RSA and pregnant groups compared to non-pregnant individuals (Wald_1_ =5.680, *p* = 0.017, Exp(B) = 1.123). Additionally, increased expression of TIGIT ICP was observed on NKT cells in RSA patients compared to non-pregnant women (Wald_1_ = 5.640, *p* = 0.018, Exp(B) = 0.950), Figure 4.

**Figure 5:**
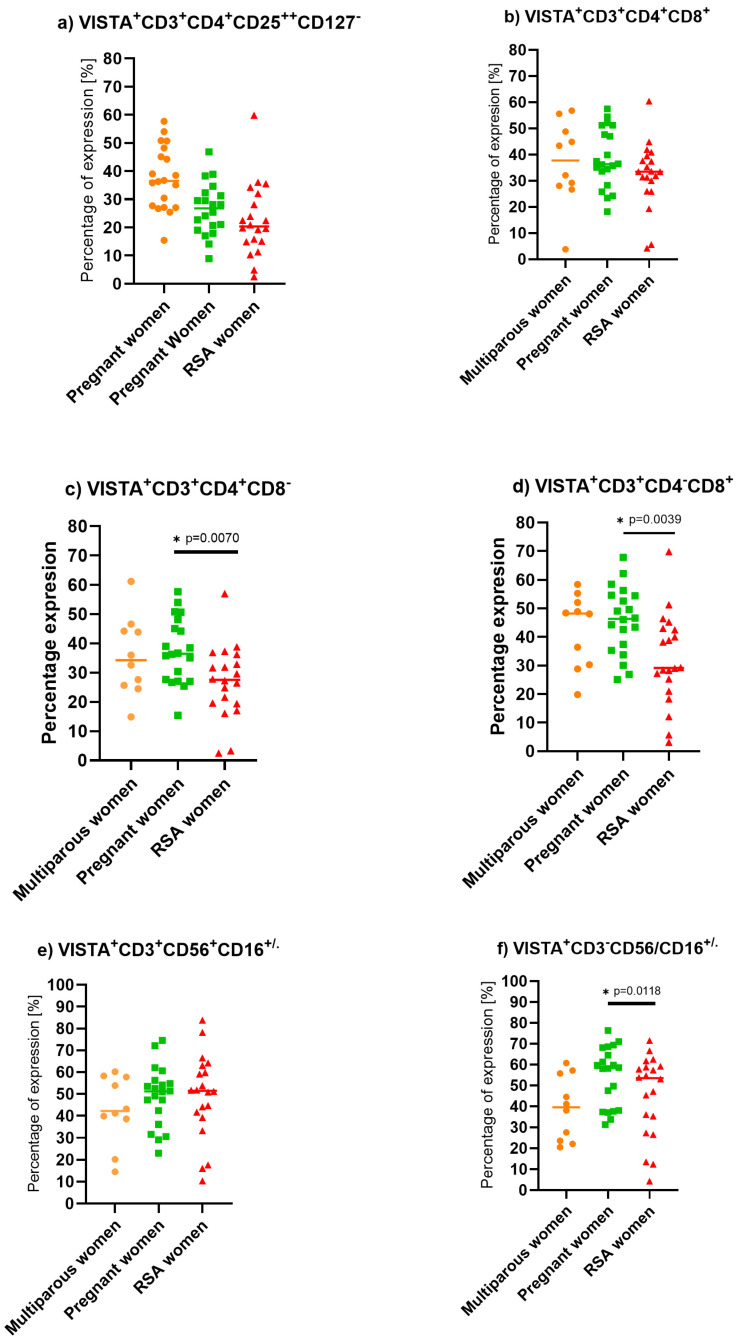
VISTA expression on isolated PBMC lymphocytes in a control group of non-pregnant multiparous women (*n* = 10), pregnant women (*n* = 20), and RSA women (*n* = 20). Statistically significant differences between groups were considered if “*p*” was below 0.05 and marked by a star. (**a**) VISTA expression on Treg cells, (**b**) VISTA expression on T lymphocytes, (**c**) VISTA expression on T helper lymphocytes, (**d**) VISTA expression on cytotoxic T lymphocytes, (**e**) VISTA expression on NKT-like cells, (**f**) VISTA expression on NK cells.

We found that pregnant women have increased expression of VISTA on NK cells compared to the non-pregnant group. Additionally, we observed decreased expression of VISTA on helper (Wald_1_ = 6.030, *p* = 0.014, Exp(B) = 1.081) and cytotoxic T lymphocytes (Wald_1_ = 7.180, *p* = 0.007, Exp(B) = 1.077) in the RSA group compared to pregnant individuals, Figure 5.

## 3. Discussion

### 3.1. Programmed Death Protein (PD-1) Expression on Lymphocytes

Data connected with PD-1 expression on lymphocytes in pregnant women are scarce. Our research showed decreased expression of PD-1 on the peripheral lymphocytes of women in the RSA group. Specifically, we found decreased expression of PD-1 on T helper cells and NKT cells when compared to multiparous women and decreased expression on T helper, cytotoxic T, T regulatory, and NKT cells when compared to pregnant women. These findings are in line with the observations of Wang et al., who showed that women with RPL have immunological differences in the percentage of peripheral lymphocytes, especially Th1, Th17, and Treg cells, and that expression of PD-1 was significantly decreased [60]. The findings suggested that women with RPL have an enhanced inflammatory immune response and decreased immune regulatory function due to altered expression of PD-1. The abnormal expression of PD-1 and PD-L1 on Th1 and Th17 cells directly involves immune imbalance at the MFI by preventing the conversion of naïve T cells into Treg cells and promoting Th17 cell survival [10,11,62].

Meggyes et al. showed, by using flow cytometry to examine the lymphocytes of healthy pregnant women, that PD-1 expression on peripheral cytotoxic and helper T lymphocytes was decreased in the first trimester compared to non-pregnant women [2]. However, in our findings, we did not confirm that data, which might have been explained by the smaller size of our multiparous group. The authors established that the cytotoxic activity of decidual PD-1^+^CD8^+^T cells compared to peripheral counterparts is decreased [2]. This difference was detected only on CD8^+^T cells, which shows the importance of inhibiting cytotoxic T cells during pregnancy. A significant decrease in the expression of inhibitory PD-1 receptors in the first and third trimesters of pregnancy could be behind the Th1 predominance in these trimesters [11]. They hypothesize that Th1 predominance could lead to an increase in PD-1 expression on CD8^+^T cells to the level seen in the second trimester. For the implementation of the PD-1-mediated inhibitory effect, the presence of the ligand molecule is also crucial. In their studies, they found a decrease in PD-L1 expression on the surface of CD4^+^ T and CD8^+^ T cells in the third trimester of pregnancy, which could affect the PD-1/PD-L1 pathway, resulting in a Th1 predominance before and during delivery [11]. Studies performed by Wang et al. showed similar results to those performed by Maygges et al. The authors also claimed that during pregnancy, PD-1 expression on peripheral lymphocytes and NK cells is suppressed [2,62].

PD-L1 expression is increased by numerous pro-inflammatory factors (LPS, GM-CSF, and VEGF) and cytokines (IFN-γ and TNF-α) [20]. It is already known that trophoblast cells present immune checkpoint ligands on their surface. Veras et al. showed high PD-L1 expression on syncytiotrophoblasts in the early term of healthy pregnancy [63]. Previous studies showed that the frequency of PD-1 expression in pregnant women’s peripheral T lymphocytes is elevated compared to non-pregnant women and soluble PD-L1 is increased throughout gestation [21,60]. PD-L1 and PL-L2 interaction with PD-1 resulted in reduced Th1 cytokine production by CD4^+^ T cells, which may be beneficial for establishing pregnancy outcomes [60,61]. The above-mentioned findings suggest that disturbances in PD-1 expression may lead to miscarriage and PD-1 plays an important role in maintaining the balance in maternal immune tolerance towards the fetus. 

### 3.2. T Cell Immunoglobulin and Mucin Domain-Containing Protein (TIM-3) Expression on Lymphocytes

We observed decreased expression of TIM-3 on studied peripheral lymphocytes, with the exception of NKT cells, in RSA women compared to pregnant women. Our findings are in line with Sun et al.’s results, who reported that in RSA patients, lowered expression of TIM-3 on peripheral NK cells might be connected with a decreased level of TGF-β in serum or with a lack of stimulus for TIM-3 [62]. Research proved that TIM-3 acts as a marker of fully mature and functional NK cells. NK cells produce high amounts of cytokines and are highly cytotoxic. Gleason showed that Galectin-9 stimulation of TIM3^+^NK cells results in the release of a large amount of IFN-γ [64]. Moreover, Ndhlovu et al. showed that NK cell-mediated cytotoxicity is suppressed after the interaction of TIM-3 with Gal-9 [65]. Meggyes et al. showed that TIM-3^+^ NK cells and TIM-3^−^ NK cells express different cytokine profiles in the peripheral blood of pregnant women compared to non-pregnant individuals. NK TIM-3^+^ cells in both non-pregnant and pregnant women have lower cytotoxic activity than NK TIM3^(−)^ [66]. Miko et al. observed that restraining the depression of TIM-3 on peripheral NK cells with increased NK cell activity is connected with the early onset of pregnancy loss [67]. Miko et al.’s findings are similar to our results, where the expression of TIM-3 on NK cells is decreased in RSA women compared to pregnant women [67]. Trophoblasts principally express Gal-9 and TIM-3 on their surfaces. Furthermore, the molecules are found in a soluble form. TIM-3/Gal-9 interaction influences both the activity and differentiation of NK cells [68]. The data suggest that trophoblasts, via TIM-3, induce the transformation of peripheral NK cells into decidual NK cells with immunoregulatory properties [66]. Hu et al. proposed that the TIM-3 signaling pathway was responsible for maintaining the anti-inflammatory phenotype of dNK cells towards the fetus [18]. Moreover, dNK cells secrete cytokines and chemokines, which are responsible for spiral artery remodeling. It is plausible that TIM-3 might have a direct or indirect influence on spiral artery remodeling [18]. Similarly, TIM-3 is expressed on decidual immune cells: CD8^+^, CD4^+^ T cells, and NK cells [69]. TIM-3 is a ligand for Galectin-9 [66,67,68,69]. IFN-γ produced by Th1 cells regulates Gal-9 expression. The interaction of TIM-3/Gal-9 prevents an excessive inflammatory immune response, initiating the anergy and apoptosis of activated T cells in the feedback loop [18]. The results of a flow cytometry experiment by Zhuang et al. showed that the ratio of CD4^+^TIM3^+^/CD4^+^ cells per PBMC was significantly higher in the URSA group than in the control group [69]. Wang et al., report that in spontaneous abortion, the number of PD-1^+^Tim-3^+^/CD4^+^ T cells is decreased, and their production of Th2-type cytokines is deficient [59]. In our study, we confirmed the declined expression of PD-1 on T, NK, and NKT cells in the RSA group. Previous observations suggested that PD-1 and TIM-3 play a role in suppressing the Th1-mediated immune response during pregnancy [70]. Studies performed in vivo in animals treated with anti-Tim-3 and anti-PD-1 antibodies showed that this treatment was highly effective in reducing proliferation and Th2-type cytokine production from decidual PD-1^+^Tim-3^+^CD4^+^ T cells in an experimental model of mouse pregnancy [71]. Blockade of Tim-3 and PD-1 caused diminished Treg function and improved Th1 and Th17 activation [71]. In contrast, the experimental results of Li et al. on RSA mice models established that manifestation of Tim-3 on T cells, as well as on placental and trophoblast cells, was higher in abortion-prone mice than in control mice [72]. Taking into consideration that TIM-3^+^ cytotoxic T lymphocytes are regarded as exhausted cells with a lack of ability to clear virus and tumor cells [18], at the maternal–fetal interface, TIM-3^+^CD8^+^T cells do not exist as exhausted cells but show high proliferative activity with an anti-inflammatory cytokine profile [73]. Moreover, Wang et al. found that the number of TIM-3^+^CD8^+^ T lymphocytes decreased in miscarriage patients [60], which coincides with our results. In addition, they report that blockade of TIM-3 in vitro results in decreased proliferation of CD8^+^ T cells and shifts their cytokine profile to release IFN-γ, which increases the cells’ cytotoxic capacity. The changes may cause trophoblast cell death. Additionally, Wang postulates that the microenvironment at the maternal–fetal interface, including the hormones, cytokines, growth factors, etc., secreted by decidual stromal cells and trophoblast cells, upregulates expression of TIM-3 on CD8^+^ and CD4^+^ lymphocytes [60]. Regulatory NKT cells represent a small proportion of decidual immune cells and may control adaptive and innate immunity [18]. Youan et al. showed that rapid and uncontrolled activation of NKT cells may lead to pregnancy loss [74]. In our study, we discover that TIM-3 expression is the highest on NKT cells in the RSA group. Li et al. hypothesize that high levels of TIM-3 lymphocytes may result in hyperimmune responses and lead to immune rejection of the fetus [72].

More and more current studies indicate the relationship of TIM-3 with the normal course of pregnancy. 

### 3.3. T Cell Immunoglobulin and ITIM Domain (TIGIT) Expression on Lymphocytes

There are only a few publications related to TIGIT expression on peripheral lymphocytes in pregnant women or women with RSA. Wang et al. proved that as the pregnancy progressed, the expression of TIGIT on NK cells gradually increased and conversely, TIGIT and PD-1 expression on T cells declined throughout pregnancy [74]. Our study showed a significant increase in TIGIT percentage in NKT cells in RSA women. Meggeys et al. presented data that TIGIT competes for the ligands (CD155 and CD112) with the activator molecule for NK and NKT cells, CD226 [11], and they showed that the percentages of CD226^+^ NK and NKT cells are increased during the first trimester of pregnancy [11]. After blocking TIGIT with an antibody, γδT cells proliferated and secreted an immunomodulatory protein, progesterone-induced blocking factor (PIBF), which facilitates immune escape and prevents embryonic rejection [7]. These results indicate that TIGIT signaling pathways are crucial for maintaining immune tolerance toward the fetus and suggest a connection with progesterone concentration Moreover, in our study, we observed decreased expression of the TIGIT molecule on Treg cells in RSA women compared to pregnant and non-pregnant women, which is in line with results obtained by Granne et al. [75]

### 3.4. V-Domain Ig-Containing Suppressor of T Cell Activation (VISTA) Expression on Lymphocytes

VISTA is a relatively newly discovered immune checkpoint, and its role and function are still not clear. There are no available data regarding VISTA expression on lymphocytes during pregnancy. We reveal that VISTA expression is decreased in RSA patients compared to pregnant women. Additionally, we find that NK VISTA expression on the lymphocytes of pregnant women is decreased compared to non-pregnant women. Some studies support the assumption that VISTA is an immune checkpoint receptor expressed on tumor-infiltrating T lymphocytes (TILs) and myeloid cells, leading to suppression of T cell activation, proliferation, and cytokine production [76,77]. VISTA functions as a ligand and receptor, conducting coinhibitory signals and suppressing T-cell activation, proliferation, and cytokine production [76]. Further studies revealed that VISTA interacts with V-set and Ig domain-containing 3 (VSIG3), which is highly expressed on placental tissue and potentially induces fetal tolerance [77]. Moreover, VISTA is involved in the regulation of Th2 cell differentiation. It upregulates the expression of Foxp3 T cells and prevents Graft versus host disease (GVHD) in an experiment in a mouse model [78,79,80]. VISTA expression on the lymphocytes of RSA patients was decreased compared to cells in pregnant women in our study, which supports this theory [79] Decreased expression of VISTA on CD4 and CD8 lymphocytes might be a reason for decreased tolerance and immunosuppressive activity in response to fetal antigens [81]. Flies et al. showed that VISTA may be crucial in the prevention of GVHD induction [82]. Flies et al. demonstrated that VISTA-/-CD4^+^ T cells show an increased effector response [83,84,85]. The features of VISTA, along with its similarity to PD-1, make VISTA a target for experimental immunotherapy for pregnancy loss. Physiological pregnancy promotes a relatively immunosuppressive environment that tolerates fetal antigens [13].

### 3.5. Lymphocyte Activation Gene-3 (LAG-3) Expression on Lymphocytes

A few ongoing studies respect the role of LAG-3 during pregnancy. Zhang et al. claim that upon engagement with MHC-Ⅱ or FGL-1, LAG-3 negatively modulates the activation, proliferation, effector function, and homeostasis of CD8^+^ and CD4^+^ T cells. LAG-3^+^Treg cells exhibit an effector-memory phenotype (CD45RA^−^CCR7^−^) with less proliferation capability and higher levels of anti-inflammatory cytokine production: IL-10 and TGF-β [86]. In early human pregnancy, LAG-3^+^Treg cells were found in the decidua and the periphery [87]. However, the difference was not established in pregnant and non-pregnant samples. Our data are the first to include LAG-3 expression on lymphocytes from women with RSA, which was significantly reduced in the entire population of lymphocytes studied, including T reg cells. In Madadi et al., studies on pre-eclampsia patients revealed lower protein levels of PD-1, LAG-3, CTLA-4, and TIM-3, as well as gene expression in decidual tissues, than in normal pregnant women [88,89].

### 3.6. Potential Clinical Applications of the Findings

#### 3.6.1. Diagnostic Tool for Immune Dysregulation in RPL

Quantifying ICP expression on lymphocytes from women with RPL could be developed as a diagnostic tool. By comparing the expression profiles of ICPs with those of healthy pregnant women, clinicians could identify immune profiles associated with higher risks of miscarriage.

Application: Flow cytometry or immunohistochemistry could be used to measure surface and intracellular ICP expression, helping to identify women at risk for RPL due to immune imbalances.

#### 3.6.2. Personalized Immunomodulatory Therapies

Once abnormal ICP expression patterns are identified, personalized therapeutic strategies could be designed to modulate immune responses. This might involve therapies that block or stimulate specific ICP pathways (e.g., PD-1/PD-L1 and TIGIT inhibitors or agonists) to restore immune tolerance and prevent fetal rejection.

Application: Immunotherapy could be tailored to the specific ICP profile of each patient, potentially using monoclonal antibodies or small-molecule inhibitors to fine-tune immune responses.

#### 3.6.3. Monitoring of Treatment Response

Tracking ICP expression during and after treatment could serve as a biomarker for therapeutic efficacy. Patients receiving immunomodulatory therapy could have their ICP levels regularly monitored to assess whether immune tolerance is being successfully restored.

Application: Serial measurement of ICP expression levels on lymphocytes throughout pregnancy could help clinicians adjust treatments dynamically, ensuring sustained immune tolerance during gestation.

#### 3.6.4. Predictive Biomarker for Pregnancy Outcomes

ICP expression could also be investigated as a predictive biomarker for pregnancy outcomes. Women with certain ICP profiles (e.g., low PD-1 or LAG-3 expression on T-cells) may be at higher risk for miscarriage. Early identification of these patients could lead to closer monitoring and early interventions during pregnancy.

Application: Pre-pregnancy screening for ICP levels might help stratify patients by risk, guiding preventive strategies or early therapeutic interventions.

### 3.7. Strengths and Limitations of This Research

This study was limited by the small number of RSA patients and multiparous and pregnant women. However, 20 women in the group were enough to obtain reliable statistical data and to confirm our findings, although further research with larger cohorts would be necessary. Although our goal was to assess a wide range of ICP expressions, recent data suggest that other ICP molecules or those lately classified as ICP molecules, e.g., OX40, also play a role in regulating fertilization, gestation, and human embryo development. A comprehensive analysis of various ICPs, including surface, soluble, and gene expression, in conjunction with cytokine correlations and the function of immune cells, could offer new insights into the issue of recurrent pregnancy loss.4. Materials and Methods

## 4. Materials and Methods

### 4.1. Materials

The fifty women participating in the study were divided into 3 groups: RSA, pregnant, and non-pregnant.

All women participating in our study were investigated regarding age, weight, height, number of miscarriages before 12 and 16 weeks of pregnancy, prodromal symptoms of pregnancy (vomiting, nausea, and breast pain), medical procedures before and during pregnancy, administration of vitamins or dietary supplements before and during pregnancy, administration of folic acid 6 weeks before pregnancy, usage of hormonal contraception, and fertility treatment. Additionally, we collected and compared data about the most common chronic diseases such as diabetes, endometriosis, insulin resistance, Hashimoto disease, and polycystic ovary syndrome. The data are presented in Table 1. 

#### 4.1.1. Non-Pregnant Multiparous Women

The control group consisted of 10 fertile, non-pregnant women without disorders in their obstetric-gynecological and internal medicine history. All women in the control group have had at least one childbirth without complication, and all subjects declared a normal course of pregnancy and delivery. None of the women in the control group had miscarriages in the past. In addition, none of them have been treated for any internal disorders.

#### 4.1.2. Pregnant Women

The pregnancy group consisted of 20 pregnant women. All patients included in the group were maximally at 12 weeks of pregnancy. Patients had ultrasonographic examinations according to Fetal Medicine Foundation standards and blood tests were performed to confirm normal course of pregnancy. 

#### 4.1.3. Recurrent Spontaneous Abortion (RSA) Women

Recurrent pregnancy loss was defined as two or more consecutive spontaneous miscarriages before the 20th week of gestation. A total of 20 patients undergoing subsequent pregnancy loss were included in the study group. All samples were collected within 72 h after miscarriage. Patients with anatomic, genetic, microbiological, immunological, and hormonal causes of abortion were excluded from the research. 

### 4.2. Sample Isolation and Preparation

Blood was collected into heparin tubes. Subsequently, blood was mixed in a 1:2 proportion by volume with phosphate-buffered saline (PBS). Then, the mixtures were laid slowly on the surface of the Histopaque-1077 (Merck & Co., Inc., Kenilworth, NJ, USA) in 15 mL centrifuge tubes and centrifuged at 800× *g* for 20 min at room temperature. The cells from the interphase were harvested and washed in PBS (Corning, NJ, USA), 600× *g*, for 10 min at room temperature. The washing step was repeated twice. Then cells were suspended in RPMI 1640 medium with stable glutamine (Biowest, Nuaillé, France) enriched with 10% heat-inactivated human serum (Merck & Co., Inc., Kenilworth, NJ, USA), antibiotic–antimycotic solution 100 I.U., penicillin, 100 µg/mL Streptomycin, and 0.25 µg/mL Amphotericin (Corning, NJ, USA).

### 4.3. Flow Cytometry Staining

After gradient isolation, 1 × 10^6^ cells were transferred to 12 × 75 mm tubes (Becton, Dickinson and Company, NJ, USA). Cells were suspended and washed in 1 mL PBS with 0.01% sodium azide (NaN_3_, Merck, NG, USA) by centrifugation at 600× *g* for 10 min at room temperature. Then, cells were suspended in 100 µL PBS with 0.01% NaN_3_, and 1st-step staining with antibodies against surface antigens was performed (Table 2). All antibodies were titrated before the experiment. Cells were stained for 15 min in the dark. After the 1st step, stained cells were washed in 1ml of PBS with 0.01% NaN_3_ and centrifuged at 600× *g* for 10 min at room temperature. After washing, cells were suspended in 400 µL 0.01% NaN_3_PBS and vortexed. Next, cells were divided equally (250 µL) into 4 FACS tubes. The 1st tube was the FMO control, the 2nd tube was additionally stained with anti-PD1 and anti-TIM-3 antibodies, the 3rd tube was stained with anti-LAG-3 antibodies, and the 4th tube was stained with anti-TIGIT and anti-VISTA antibodies (Table 3). Then, cells were incubated for 15 min in the dark at room temperature. Finally, cells were washed with 1 mL of 0.01% NaN_3_PBS and centrifuged at 600× *g* for 10 min at room temperature. After washing, cells were suspended in 150 µL of 0.01% NaN_3_PBS and prepared for acquisition. Acquisition and data analysis were performed with the flow cytometry method (FACS Canto II) and Diva 6.0 software, Figures SD1 and SD2 (please refer to Supplementary Data). 

### 4.4. Statistical Analysis

Based on data from the literature indicating that the expression of immune-regulating molecules in the general population ranges between 40–60%, we assumed an average expression of Mi1 = 50%. Power analysis for the hypothesis that the mean expression Mi2 in the population of women with recurrent miscarriages falls between 30–50% (Mi2 = 40%) was conducted. The alternative hypothesis assumes that Mi1 ≤ Mi2, with a significance level (alpha error) of 0.05 and a standard deviation (Sigma) of 15%. For the predicted test power of 0.8, the sample size N was calculated to be 20 individuals per group. Therefore, a sample size of 20 individuals per group seems justified given the pilot nature of the project.

Statistical analyses were performed with Graph Pad Prism 8.4.1 GraphPad Software, Boston, MA, USA and the results were shown as mean plus/minus the standard deviation (SD). The Gaussian distribution was determined with the Shapiro–Wilk test. The analyses between sets of data with Gaussian distribution were performed by considering the F test. For groups with the same SD, an unpaired *t*-test was used. For groups with different SDs, an unpaired T-test with Welch’s correction was used. The analyses between groups without Gaussian distribution were performed with the Mann–Whitney test. *p*-values below 0.05, (*p* < 0.05) were considered as statistically significant and marked on the graphs as *—a star.

The odds ratio was calculated using SPSS 29 software for multiparameter logistic regression, where Group A represents RSA patients and Group B represents the control groups (including both pregnant and non-pregnant women). The odds ratio is reported as Exp (B), along with the Wald statistic for 1 degree of freedom (df) and the corresponding statistical significance. The odds ratio was given with a 95% confidence interval.

## 5. Conclusions

The provided data indicate immunological differences between women during pregnancy, after miscarriage, and in multiparous women. The most remarkable features of lymphocytes in RSA women were diminished expression of PD-1 and LAG-3 on T (Th and Tc), Treg, NK, and NKT cells and impaired expression of TIM-3 and VISTA on CD8 cytotoxic T and NK cells, with accompanying increased expression of TIGIT and TIM-3 on NKT cells. Our analysis confirmed the magnitude of the roles of PD-1 and TIM-3 in RSA and particularly emphasizes the role of LAG-3. One should not underestimate the role of TIGIT and VISTA in impairing the regulatory activity of CD8 cytotoxic and NKT cells. Future research on decidual immune cells concerning the above-mentioned ICPs will add further insight into the role of biomolecules in recurrent pregnancy loss. 

## Figures and Tables

**Table 1:** Characteristic of the studied groups according to Age, BMI, internal disease, and supplementation.

	Median and Q1–Q4 Quartile	Non-Pregnant Multiparous Women	Pregnant Women	RSA Women	*p*-Value ^1^ RSA vs. Pregnant ^2^ RSA vs. Multiparous ^3^ Multiparous vs. Pregnant
Age	Median	33.5	30	34	^1^ *p* = 0.26
	Q1	26	25	22	^2^ *p* = 0.26
	Q4	40	39	40	^3^ *p* = 0.4
BMI (body mass index)	Median	25	21.6	21.8	^1^ *p* = 0.5
	Q1	18.6	16.7	17.9	^2^ *p* = 0.48
	Q4	36.2	31.4	37.2	^3^ *p* = 0.28
Number of full-term pregnancies	Median	2	1	0	^1^ *p* = 0.027 *
	Q1	1	1	0	^2^ *p* = 0.00001 **
	Q4	3	3	0	^3^ *p* = 0.007 *
Number of miscarriages	Median	0	0	3	^1^ *p* = 0.0001 **
	Q1	0	0	2	^2^ *p* = 0.0001 **
	Q4	0	0	5	^3^ *p* = 0.5
Pregnancy duration (weeks)	Median	N/A	12.8	8.8	^1^ *p* = 0.1
	Q1	N/A	11.7	4.0	^2^ N/A
	Q4	N/A	14.6	13.6	^3^ N/A
The occurrence of chronic diseases					
		Non-Pregnant multiparous women	Pregnant women	RSA women	
Diabetes		0%	10%	5%	
Endometriosis		0%	0%	0%	
Insulin resistance		1%	5%	5%	
Hashimoto disease		0%	0%	0%	
Polycystic ovary syndrome		0%	10%	0%	
Diet supplements and folic acid administration before pregnancy and during pregnancy					
Folic acid administration		60%	65%	80%	
Medicine and dietary supplement administration before pregnancy		30%	60%	75%	
Salicylic acid and dietary supplement administration during pregnancy		40%	90%	75%	
No medicine and dietary supplement administration before pregnancy		70%	40%	25%	
No medicine and dietary supplement administration during pregnancy		60%	10%	25%	

Data collected from the questionnaire completed by the participants in this study. Number of participants in the group: non-pregnant multiparous women (*n* = 10), pregnant (*n* = 20), RSA (*n* = 20). *p*-values statistically significant below 0.05 (*p* < 0.05) were marked as *, and *p* < 0.001 was marked as **. N/A: not applicable. ^1^ RSA vs. Pregnant, ^2^ RSA vs. Multiparous, ^3^ Multiparous vs. Pregnant. Data are presented as median and 25th (Q1)–75th (Q4) percentile, or percentage of the group.

**Table 2:** Antibodies used for first-step staining and the FMO control.

Antibody	Fluorochrome	Clone	Volume after Titration	Manufacturer
Anti-CD3	PreCP	SK7	2 µL	Becton Dickinson
Anti-CD4	APC-Cy7	RPA-T4	0.5 µL	Becton Dickinson
Anti-CD8	APC	SK1	0.5 µL	Becton Dickinson
Anti-CD25	FITC	CD25-4E3	1 µL	Becton Dickinson
Anti-CD127	BV450	HIL-7R-M21	0.5 µL	Becton Dickinson
Anti-CD56	PE-Cy7	B159	1 µL	Becton Dickinson

**Table 3:** Antibodies used for second-step staining.

Experiment	Antibody	Fluorochrome	Clone	Volume after Titration	Manufacturer
1st tube	-	-	-	-	-
2nd tube	Anti-PD1	BV480	EH12.1	1 µL	Becton Dickinson
	Anti-Tim3	PE	7D3	0.5 µL	Becton Dickinson
3rd tube	Anti-Lag3	BV480	T47-530	1 µL	Becton Dickinson
4th tube	Anti -TIGIT	PE	741182	1 µL	Becton Dickinson
	Anti-VISTA	BV480		1 µL	Becton Dickinson

## Data Availability

The sICP.xlsx data used to support the findings of this study are available from the corresponding author upon request.

## Supplementary Materials

The following supporting information can be downloaded at: https://www.mdpi.com/article/10.3390/ijms25179378/s1.

## Abbreviations

APC—antigen-presenting cells, CD3—cluster of differentiation of lymphocytes T, CD4—cluster of differentiation helper T lymphocytes, CD8—cluster of differentiation of cytotoxic T lymphocytes, CD16—cluster of differentiation of NK lymphocytes, FcγRIII receptor for IgG type 3, CD56—NCAM—neural cell adhesion molecule, CD127—Interleukin-7 receptor subunit alpha, CTLA-4- cytotoxic T cell antigen 4, FMO—fluorescence minus one, ICP—Immune checkpoints, LAG-3—Lymphocyte-activation gene 3, IFN-γ—interferon-γ, MFI—maternal-fetal interface, NK cells—Natural Killer cells, NKT—Natural Killer T lymphocyte, PBMC—peripheral blood mononuclear cells, PBS—phosphate buffered saline, PD-1—programmed cell death protein 1, PD-L1—programmed cell death ligand 1, PD-L2—programmed cell death ligand 2, RSA—recurrent spontaneous abortion, TCR—T cell receptor, Th1—T helper lymphocyte 1, Th2—T helper lymphocyte 2, TIGIT-T cell immunoglobulin and ITIM domain, TIM-3—T cell immunoglobulin-3, TNF-α—tumor necrosis factor-α, VISTA—V-domain Ig suppressor of T cell activation.
