# Supplementary material for: Surface Immune Checkpoints as Potential Biomarkers in Physiological Pregnancy and Recurrent Pregnancy Loss
Source: Int J Mol Sci. 2024 Aug 29;25(17):9378. doi: 10.3390/ijms25179378 (PMC11395075; doi:10.3390/ijms25179378)
Supplement: Supplementary file 1 [file ijms-25-09378-s001.zip › Supplementary data-Surface ICPs.pdf]

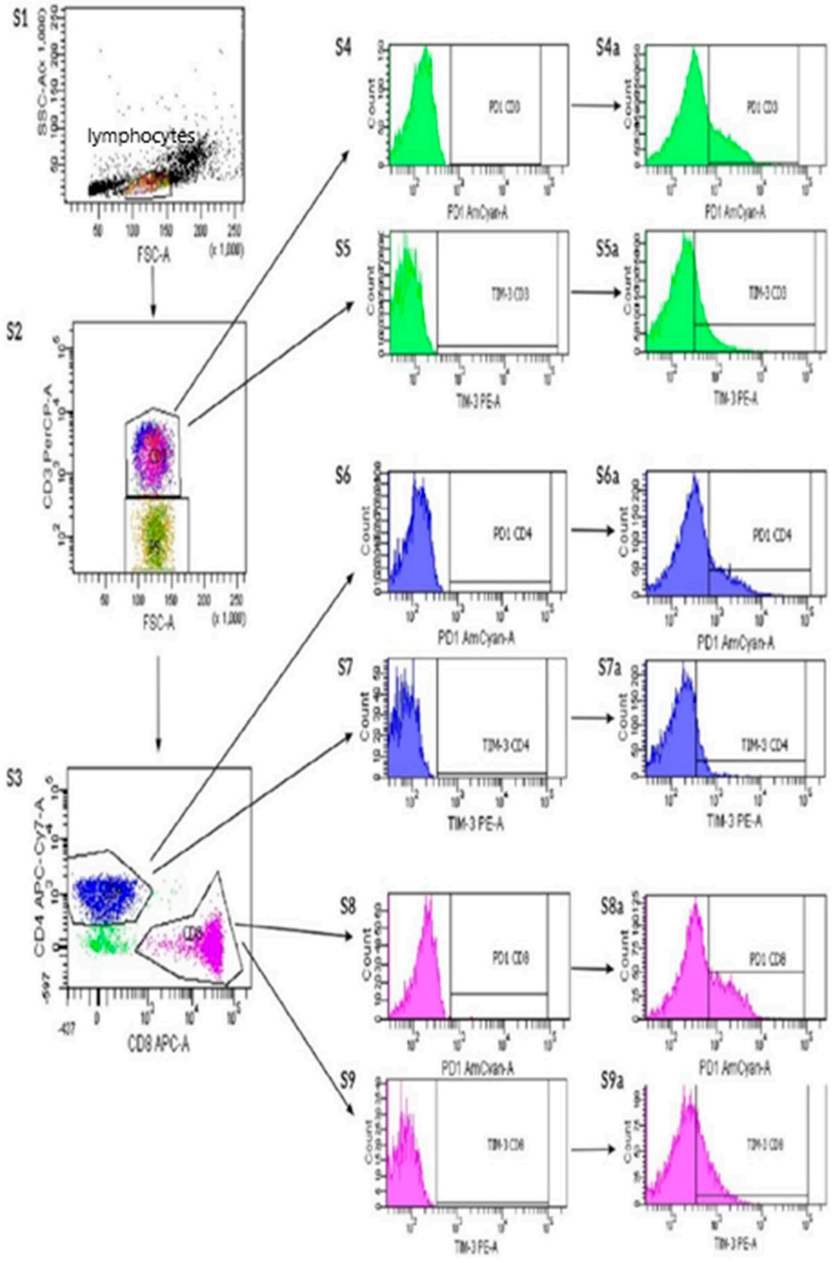

Fig.SD1. Lymphocyte T cytometric analysis - scheme. The PE channel was used to label TIM-3 and TIGIT molecules, depending on the tube, and the AmCyan channel was used to label PD1, LAG-3 and VISTA molecules, S1 - gate for lymphocytes based on SSC and FSC dot-plot, S2-CD3-T cells gate based on PerCP fluorescence/FSC-A dot plot, S3- gates for T helper cells and cytotoxic T lymphocytes based on CD4-APC-Cy7 and CD8-APC fluorescence dot plot, S4-S5-histogram of FMO controls for PD-1 and TIM-3 on CD3 cells based on PE fluorescence, S6-S7 FMO controls

for PD-1 and TIM-3 on CD4 cells based on negative AmCyan/PE fluorescence, S8-S9- FMO control for PD-1 and TIM-3 on CD8 cells, S4a - expression of PD-1 on CD3 cells above FMO AmCyan fluorescence, S5a - expression of TIM-3 on CD3 cells above FMO-PE fluorescence , S6a - expression of on PD-1 on CD4 cells above FMO-AmCyan fluorescence, S7a - expression of TIM-3 on CD4 cells above FMO-PE fluorescence, S8a - expression of PD-1 on CD8 cells based on FMO control and AmCyan fluorescence, S9a - expression of TIM-3 on CD8 cells based on FMO control and PE fluoresvence; S-sample, CD4-T helper cells, CD8-Cytotoxic T cells, CD3-lymphocytes T.

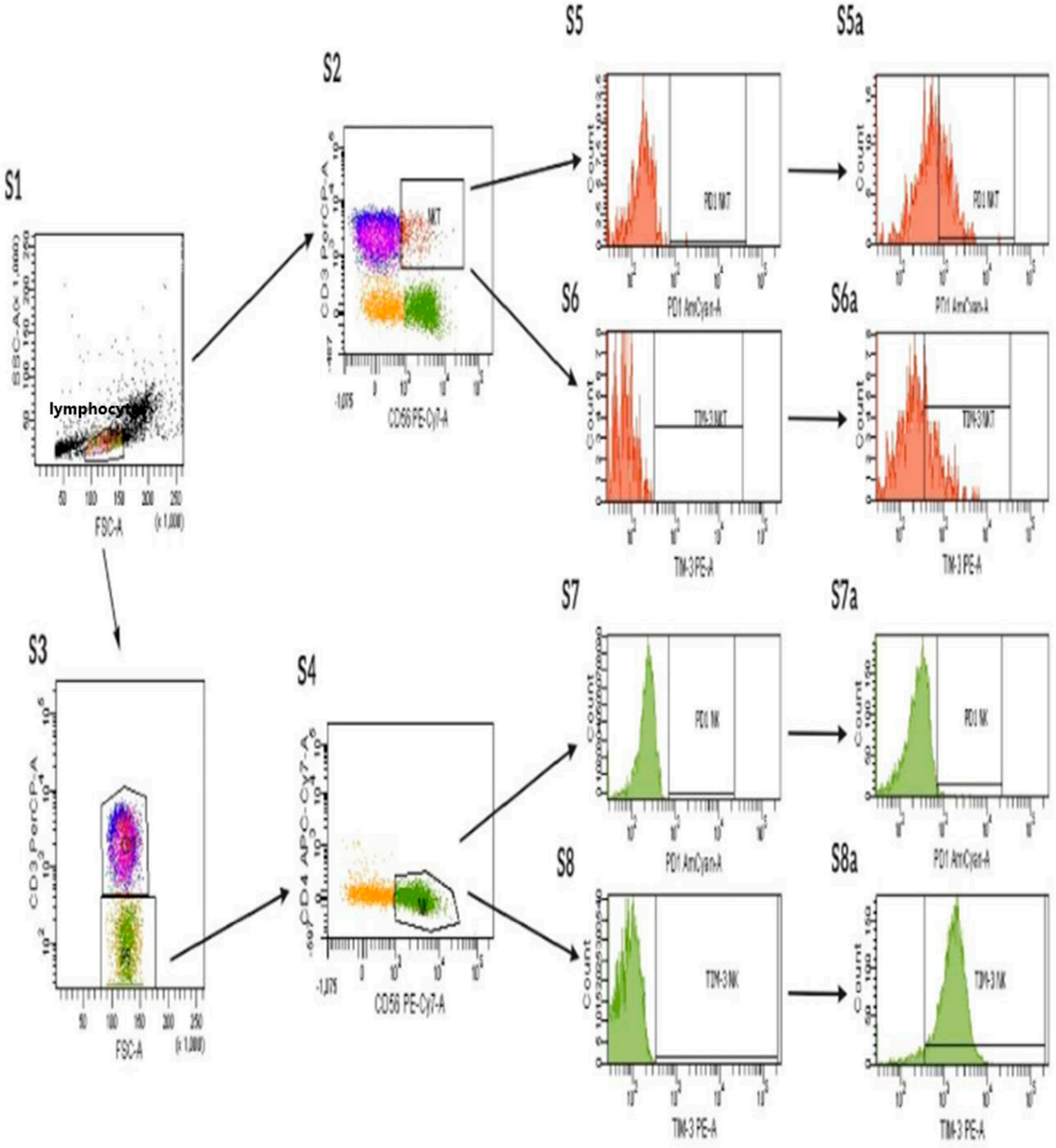

Fig.SD2. Cytometric analysis of NK and NKT cells - scheme. The PE channel was used to label TIM-3 and TIGIT molecules, depending on the tube, and the AmCyan channel was used to label PD1, LAG-3 and VISTA molecules, S1 - gate for lymphocytes based on SSC and FSC dot-plot, S2 - gate for NKT cells based on positive CD3-PerCP and CD56-PeCy7 fluorescence dot plot, S3- CD3-PerCP negative fluorescence for CD3 of cells gate, S4 - gate for NK cells based on CD4-APC-Cy7 and CD56-PE-Cy7 fluorescence among CD3 negative cells, S5 - histogram of FMO-negative for AmCyan fluorescence - control for PD-1 on NKT cells, S5a - histogram for PD-1 on

NKT cells based on fluorescence above FMO control, S6 - histogram of FMO control-negative for PE fluorescence - for TIM-3 on NKT cells, S6a - histogram for TIM-3 on NKT cells based on fluorescence above FMO control, S7 - histogram of FMO control for PD-1 on NK cells, S7a - histogram for PD-1 on NK cells based on fluorescence above FMO control, S8 - histogram of FMO control for TIM-3 on NK cells, S6a - histogram for TIM-3 on NK cells based on fluorescence above FMO control;
